# BCSLinker: automatic method for constructing a knowledge graph of venous thromboembolism based on joint learning

**DOI:** 10.3389/fmed.2024.1272224

**Published:** 2024-05-09

**Authors:** Fenghua Cai, Jianfeng He, Yunchuan Liu, Hongjiang Zhang

**Affiliations:** ^1^Faculty of Information Engineering and Automation, Kunming University of Science and Technology, Kunming, Yunnan, China; ^2^Department of Medical Imaging, The First People Hospital of Anning City, Anning, China

**Keywords:** venous thromboembolism, knowledge graph, joint entity and relation extraction, Chinese electronic medical records, deep learning

## Abstract

**Background:**

Venous thromboembolism (VTE) is characterized by high morbidity, mortality, and complex treatment. A VTE knowledge graph (VTEKG) can effectively integrate VTE-related medical knowledge and offer an intuitive description and analysis of the relations between medical entities. However, current methods for constructing knowledge graphs typically suffer from error propagation and redundant information.

**Methods:**

In this study, we propose a deep learning-based joint extraction model, Biaffine Common-Sequence Self-Attention Linker (BCSLinker), for Chinese electronic medical records to address the issues mentioned above, which often occur when constructing a VTEKG. First, the Biaffine Common-Sequence Self-Attention (BCsSa) module is employed to create global matrices and extract entities and relations simultaneously, mitigating error propagation. Second, the multi-label cross-entropy loss is utilized to diminish the impact of redundant information and enhance information extraction.

**Results:**

We used the electronic medical record data of VTE patients from a tertiary hospital, achieving an F1 score of 86.9% on BCSLinker. It outperforms the other joint entity and relation extraction models discussed in this study. In addition, we developed a question-answering system based on the VTEKG as a structured data source.

**Conclusion:**

This study has constructed a more accurate and comprehensive VTEKG that can provide reference for diagnosing, evaluating, and treating VTE as well as supporting patient self-care, which is of considerable clinical value.

## 1 Introduction

Venous thromboembolism (VTE) encompasses a spectrum of diseases, including Deep Vein Thrombosis (DVT) and Pulmonary Embolism (PE) (1), and its mortality is only second to cancer and myocardial infarction (2). Nevertheless, only a minority of patients have received the recommended medical treatment for VTE, which is even worse in remote areas (3). Thus, there is an urgent need to enhance the ability of non-specialists to treat VTE in a timely manner and raise patients’ awareness of VTE risks.

Electronic medical records document patients’ comprehensive medical activities in hospitals and serve as a rich source of specialized medical knowledge. Knowledge graphs can effectively integrate medical knowledge from electronic medical records, improve the organization and management of medical knowledge, and support physicians in diagnosis while popularizing relevant medical knowledge to patients (4–6), which offers an alternative approach to improving the timely diagnosis and treatment of VTE by non-specialists and raises patients’ awareness of VTE risks. However, electronic medical record text data typically exists in an unstructured format, making it hard to extract helpful knowledge for constructing a knowledge graph. Information Extraction (IE) is capable of identifying specific named entities and relations from unstructured electronic medical records closely related to the patients, yielding valuable medical knowledge (7–9). Nonetheless, traditional methods for extracting information from electronic medical records depend on factors such as specific domains, languages, and text styles, resulting in limited system portability. Furthermore, these methods make it difficult to enumerate all the rules that need to be modeled (10).

Deep learning has been utilized to classify relations between medical entities in electronic medical records (11, 12), minimizing dependence on manual feature engineering and addressing issues associated with traditional information extraction methods. Among the deep learning-based information extraction techniques, pipeline approaches treat named entity recognition and relation classification as two distinct subtasks (13–15). It first performs named entity recognition followed by relation classification based on the results of named entity recognition. However, these approaches overlook the inherent connection between the two subtasks, and error propagation in named entity recognition can adversely impact the accuracy of relation classification (16).

To address these challenges, Miwa et al. (16) initially introduced a joint extraction model for entities and relations to obtain entities and relations between them through a unified architecture. Existing research typically divides joint extraction into several basic modules or subtasks to simplify complex tasks. Wei et al. (17) introduced CasRel, which first identifies all potential subjects in a sentence and then applies relation-specific taggers for each subject to detect all possible relations and corresponding objects. Zheng et al. (18) presented PRGC, decomposing joint extraction of entities and relations into three subtasks: Relation Judgment, Entity Extraction, and Subject-object Alignment to extract relations between entities in a stepwise manner. BiRTE (19) extracts all possible entity pairs from both directions and assigns all potential relations to each entity pair using a biaffine model. With the inherent connection between subtasks considered, these multi-module multi-step extraction methods use distinct modules and interrelated steps of processing to extract entities and relations sequentially. However, they remain susceptible to error propagation (20).

Multi-module one-step extraction methods address error propagation in joint models by extracting entities and relations at one time and combining them into triples. SPN (21) transforms joint entity and relation extraction into a set prediction problem and combines non-autoregressive parallel decoding with a bipartite matching loss to address the relational triples prediction issue. Shang et al. (22) presented OneRel, employing a score-based classifier to assess whether a token pair and a relation constitute a relational triple. GRTE (23) generates a table feature for each relation, explores the global association between the relations and token pairs, and integrates these into each relation’s table feature to extract relational triples. Huang et al. (24) combined BERT with a multi-head selection model and added soft label embedding to enhance the information extraction capabilities of the model. While these multi-module one-step extraction methods avoid error propagation, it suffers from excessive redundant information.

Consequently, we propose a joint extraction model of entities and relations, Biaffine Common-Sequence Self-Attention Linker (BCSLinker), for constructing a VTE knowledge graph (VTEKG). First, the Biaffine Common-Sequence Self-Attention (BCsSa) module is proposed to extract common features in the electronic medical record dataset utilizing the common-sequence self-attention mechanism. Additionally, the information interaction between medical entities in the electronic medical record text data is enhanced by a biaffine model, constructing global matrices and extracting entities and relations simultaneously to avoid error propagation. Second, the multi-label cross-entropy loss is employed to mitigate the impact of redundant information generated in the model and improve the information extraction. Finally, we construct the VTEKG using specialized medical knowledge extracted from the electronic medical records and develop a prototype of a question-answering system based on the VTEKG. Furthermore, we conduct experiments on the system and analyze the results.

## 2 Materials and methods

### 2.1 Data

The experimental data in this study, encompassing chief complaints, past medical history, test results, diagnoses, preoperative assessments, postoperative evaluations, and treatment plans, were sourced from the electronic clinical medical records of a tertiary hospital in Yunnan province. Access to the medical records was granted through patient consent and approved by the Ethics Committee. We collected 16,000 electronic medical records, from which 1,600 were selected based on whether there are risk factors associated with VTE. Data cleaning was conducted initially, including removing duplicate data, missing values, and outliers. After an extensive review of labeling specifications, we employed the labeling specifications provided by the Network Intelligence Laboratory of Harbin Institute of Technology University (25) to classify entities in the electronic medical records into diseases, symptoms, tests, and treatments. To accommodate the characteristics of VTE, we established additional entity labels based on the table attributes in the VTE-related risk assessment scale, as well as focusing on coagulation, liver function, and other relevant test indicators that can help further prompt physicians for VTE diagnosis and treatment. The entity types displayed in Table 1.

**Table 1:** Entity types of the dataset.

**Entity type**	**Meaning**	**Example**
Disease	The name of the disease	Hypertension
Symptom	Patient’s discomfort or unusual sensation	Lower extremity edema
Drug	The drug used for treatment	Warfarin
Factor	The cause of a symptom or disease	Bedridden
Treatment	Treatments other than drugs	Arthroscopy surgery
Test	Medical examination items	Electrocardiography
Matter	Notes for patients	Limb elevation

Entity relations are classified based on entity types. We established 14 types of relations between entities upon the above-mentioned named entity types. The 14 relation types are displayed in Table 2. Subsequently, a professional doctor with 5 years of experience conducted manual labeling and another doctor with 10 years of experience performed reviews.

**Table 2:** Relation types of the dataset.

**Relation type**	**Specific relations between entities**	**Symbol**
Treatment-disease	The treatment of the disease	TrAD
Treatment-symptom	The treatment of the symptom	TrAS
Test-disease	The test confirmed the disease	TeRD
Test-disease	Because the disease takes the test	TeBD
Test-symptom	The test revealed symptoms	TeRS
Test-symptom	Because the symptom takes the test	TeAS
Disease-symptom	The disease causes symptoms	DIS
Symptom-disease	Symptoms diagnosed as a disease	SDD
Factor-symptom	Factors contributing to symptoms	FCS
Drug-disease	The drug is used to treat diseases	DrAD
Drug-symptom	The drug is used to treat symptoms	DrAS
Drug-treatment	The drug as a method of treatment	DrTr
Disease-matter	Advisories and precautions for the disease	DM
Symptom-matter	Advisories and precautions for the symptom	SM

After multiple iterations, 1,600 electronic medical records were labeled, with 9,977 sentences and 6,011 relational triples. The 1,600 electronic medical records were divided into three data sets - training, validation, and test, with a ratio of 8:1:1. The distribution of relation quantities among the three data sets is presented in Table 3.

**Table 3:** Dataset relation statistics.

**Relation type**	**Training set**	**Validation set**	**Test set**
TrAD	395	43	64
TrAS	1,406	217	207
TeRD	326	30	32
TeBD	151	19	19
TeRS	148	16	20
TeAS	706	121	103
DIS	271	33	23
SDD	188	24	29
FCS	95	16	18
DrAD	258	32	27
DrAS	269	28	33
DrTr	296	30	46
DM	88	10	10
SM	109	13	15

### 2.2 Methods

**Figure 1:**
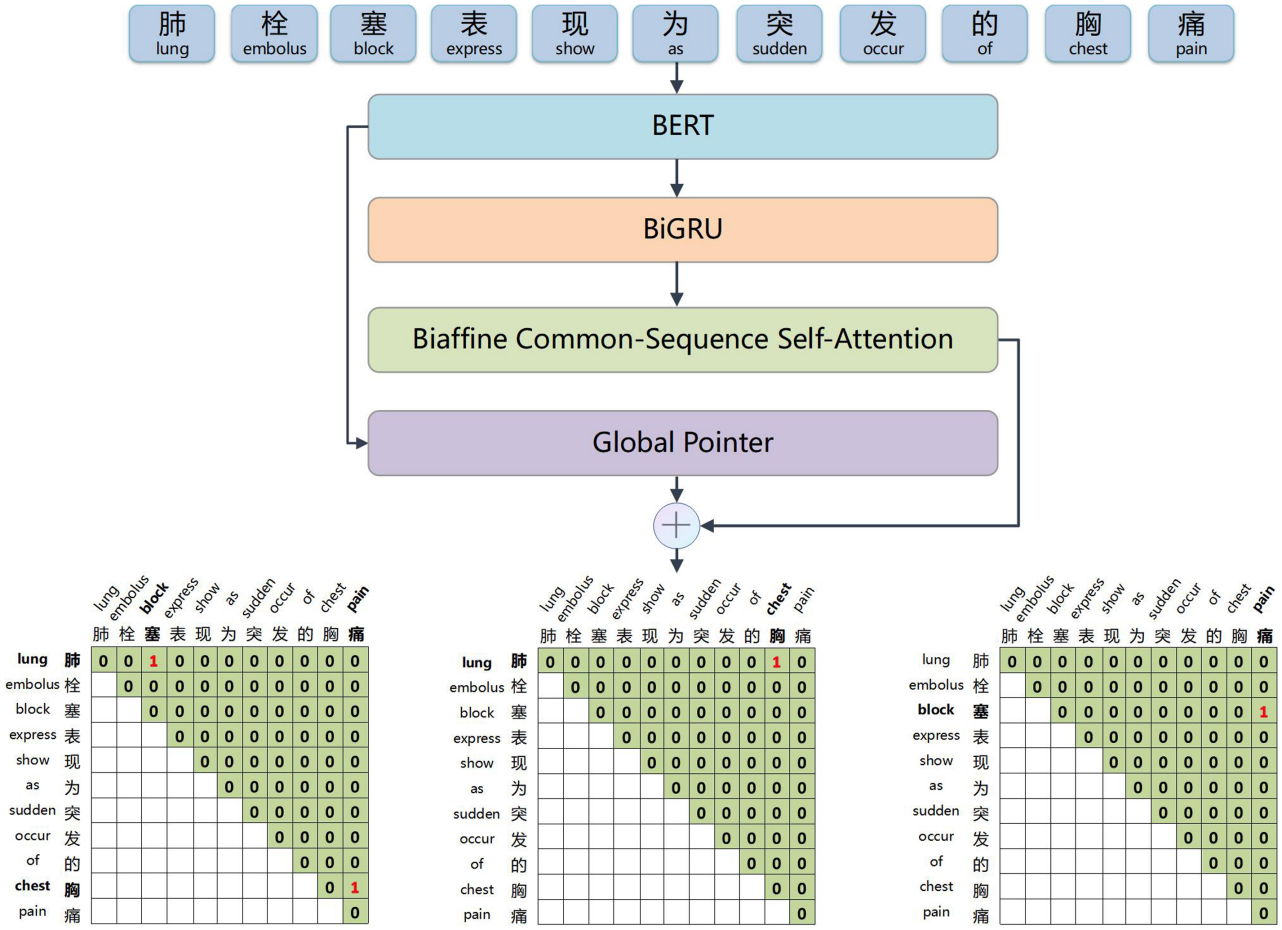
Example of the overall structure of BCSLinker. The input sequence is ‘肺栓塞表现为突发的胸痛’ (The common symptoms of a pulmonary embolism include sudden chest pain), and the output sequence corresponds to global matrices.

This paper proposes BCSLinker to extract VTE-related entities and relations from Chinese electronic medical records for constructing a VTEKG. The model comprises a BERT word embedding layer (26), a BiGRU context feature extraction layer (27), a Global Pointer layer (28), and the BCsSa layer. Figure 1 illustrates the overall structure of BCSLinker. First, the BERT word embedding layer transforms the input electronic medical record text into corresponding word vectors. Then, the word vectors acquire contextual semantic information through the BiGRU context feature extraction layer. Third, the BCsSa module constructs global matrices by incorporating common features of the electronic medical record dataset into contextual semantic information, enabling entity information interaction. The global matrices and global features obtained through the Global Pointer layer are fused to generate scoring matrices for entity recognition and relation extraction. Finally, we employ the multi-label cross-entropy loss (28) to mitigate the impact of redundant information generated in the model.

#### 2.2.1 BERT word embedding layer

**Figure 2:**
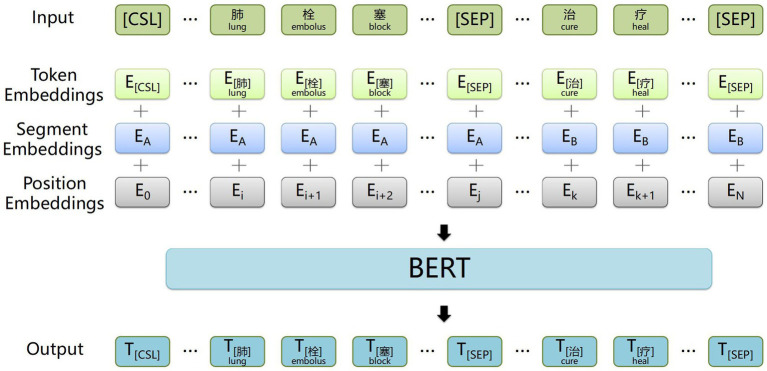
Example of input and output of the BERT word embedding layer. The input sequence is ‘考虑肺栓塞，予抗凝药物治疗’ (Suspect a pulmonary embolism, treat with anticoagulants), and the output is sequence corresponds word vector.

The BERT word embedding layer, depicted in Figure 2, transforms the electronic medical record text into word vectors that the neural network model can recognize and train effectively. This weight-efficient network is obtained through pre-training on a large-scale text corpus. It allows for dynamic optimization for specific tasks and requires only fine-tuning with the small electronic medical record dataset, thus reducing the difficulty of medical information extraction tasks.

#### 2.2.2 BiGRU context feature extraction layer

**Figure 3:**
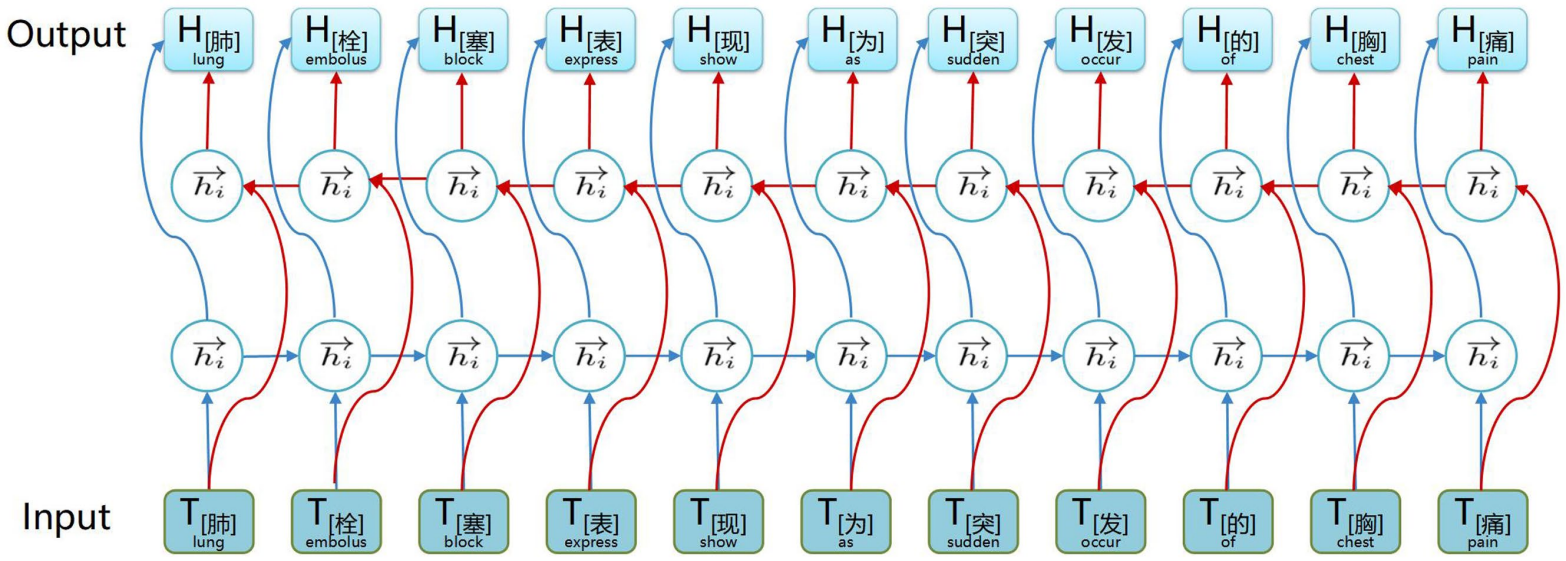
Example structure of the BiGRU contextual feature extraction layer. The input is the word vectors corresponding to ‘肺栓塞表现为突发的胸痛’ (The common symptoms of a pulmonary embolism include sudden chest pain), and the output is contextual semantic information.

The structure of the BiGRU context feature extraction layer is illustrated in Figure 3. A BiGRU unit, consisting of a forward GRU unit and a backward GRU unit, extracts both forward and backward information from words in the electronic medical record text data, which enables the acquisition of semantic features and long-distance information while avoiding vanishing and exploding gradients. Additionally, compared with other models, the GRU model features relatively fewer parameters, which helps prevent overfitting and enhances training speed, especially when dealing with a small electronic medical record dataset.

#### 2.2.3 BCsSa layer

**Figure 4:**
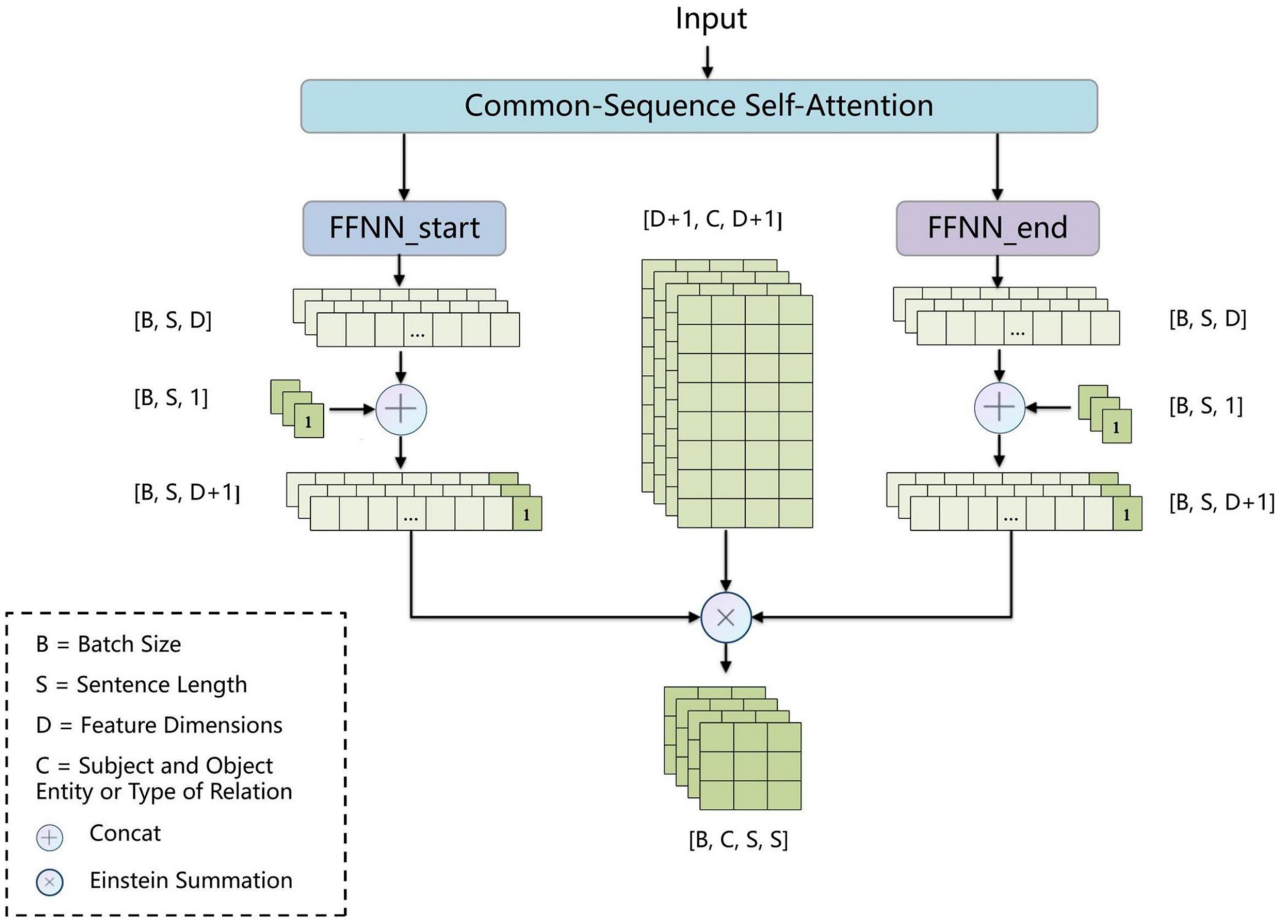
Flowchart of the BCsSa layer.

To address error propagation, we propose the BCsSa module, which constructs global matrices and simultaneously extracts entities and relations. The process of this module is depicted in Figure 4. First, the common-sequence self-attention mechanism captures global information and common features from the contextual semantic information. Second, the obtained features are fed into two feedforward neural networks to acquire two different features representing the links between entity head and entity tail, subject head and object head, and subject tail and object tail. Lastly, a biaffine model (29) facilitates interaction between entity pairs in the electronic medical record text data, improving entity recognition and relation extraction. By constructing global matrices for identifying entities and relations between them, entity recognition and relation extraction are accomplished simultaneously in a single step, thereby avoiding error propagation.

##### 2.2.3.1 BCsSa module

**Figure 5:**
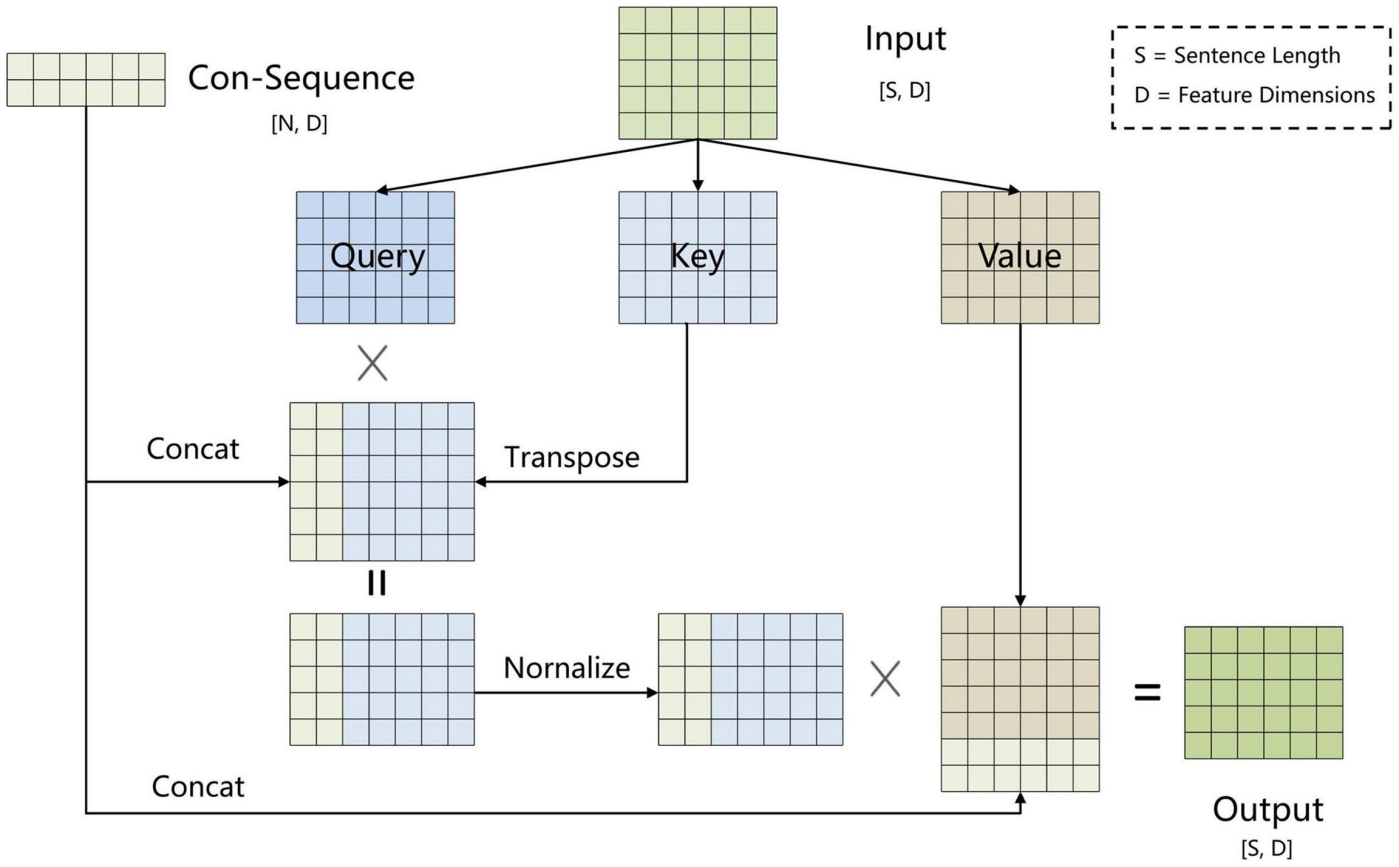
Structure of the common-sequence self-attention.

All electronic medical record text data in this paper is from the same hospital and contains entities and relations related to VTE, so common features exist among the data. To capture these common features, we propose an improved self-attention mechanism that allows the neural network to capture long-term dependencies by computing correlations between every two positions in the text while also capturing additional common features of the electronic medical record text. Figure 5 illustrates the improved self-attention structure where we establish an input-independent learning matrix jointly trained by all input data. The matrix captures common features of the input data and is added to the self-attention mechanism for operation.

For the input sequence $X=\left\{x_{1},x_{2},\ldots ,x_{n}\right\}$ , where $x_{i}\in R^{d}$ represents the embedded vector at the position i in the input sequence, three separate linear transformations are applied to transform the input embedding X into query, key and value vectors Q , K , V . The specific calculations are as Equations (1–3).

(1) $$Q=XW_{Q}$$

(2) $$K=XW_{K}$$

(3) $$V=XW_{V}$$

Where $W_{Q}W_{K}W_{V}$ are d×d weight matrices that need to be learned.

To obtain common features of the electronic medical record dataset, we set up a learnable shared matrix C with input-independent n×d , where n is a hyperparameter. The shared matrix C is incorporated into the key vector K via equation (4) to obtain $K_{C}$ , which is matched with the query vector Q to calculate the correlation between the two. The higher the correlation is, the greater the weight corresponding to $V_{C}$ will be. The specific equations are as Equations (4–6).

(4) $$K_{C}={\left[\begin{array}{c} K\\ C \end{array}\right]}^{T}$$

(5) $$V_{C}={\left[\begin{array}{c} V\\ C \end{array}\right]}^{T}$$

(6) $$\mathrm{output}=\mathrm{softmax}\left(\frac{QK_{C}}{\sqrt{d_{k}}}\right)V_{C}$$

Where $d_{k}$ is the dimension of the key vector K . Converting the attention matrix to a normal distribution through $\sqrt{d_{k}}$ makes the structure stable while balancing the back-propagation gradients.

Inspired by the dependency parsing model of Dozat and Manning (29), for sequences that contain common features of the electronic medical record dataset, we used two independent feedforward neural networks to create different representations ( $h_{s}$ and $h_{s}$ ) for the beginning and end of the span. The three types of global matrices are used to represent entity head and entity tail, subject head and object head, and subject tail and object tail, respectively, enabling the model to learn these different features separately and improve the precision of the extraction results. We use the biaffine model to create global matrices $g_{m}$ of c×s×s , where c is the subject and object entity or type of relation and s is the sentence length. We calculate the score for each span i by Equations (7–9).

(7) $$h_{s}\left(i\right)=FFNN_{s}\left(\alpha_{s_{i}}\right)$$

(8) $$h_{s}\left(i\right)=FFNN_{s}\left(\alpha_{s_{i}}\right)$$

(9) $$g_{m}\left(i\right)=h_{s}{\left(i\right)}^{T}U_{m}h_{e}\left(i\right)+b_{m}$$

Where α is the word representation, $s_{i}$ and $e_{i}$ denote the start and end indices of span i , $U_{m}$ is a learnable $\left(d+1\right)\times c\times \left(d+1\right)$ tensor and $b_{m}$ is the bias.

##### 2.2.3.2 Global matrix

**Figure 6:**
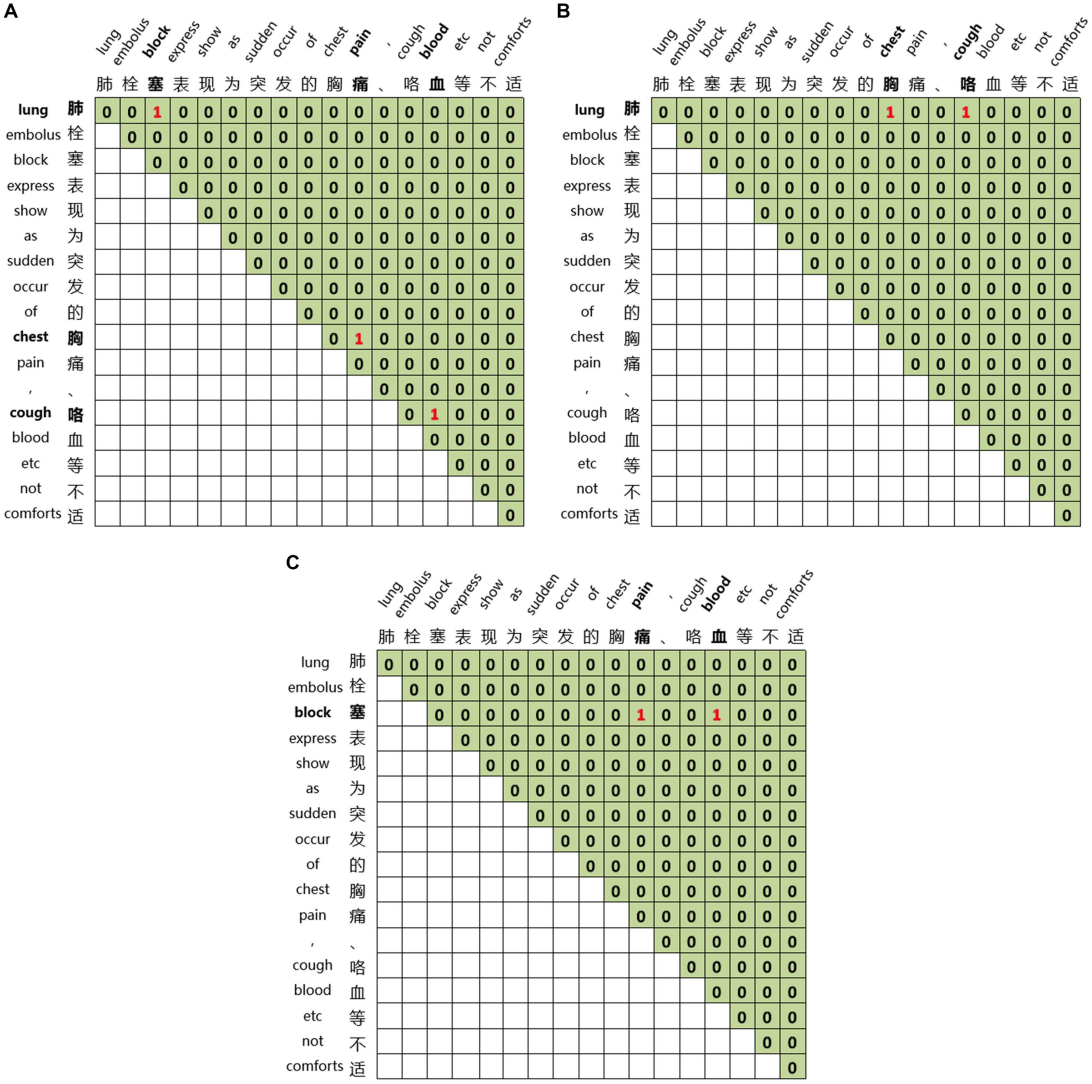
Global matrices. **(A)** The link between the head and tail of the entities. **(B)** The link between the subject’s head and the objects’ head. **(C)** The link between the subject’s tail and the objects’ tail.

Global matrices are employed for joint entity and relation extraction. We construct three global matrices for an input sequence to indicate the links between entity head and entity tail, subject head and object head, subject tail and object tail, respectively. For the sentence ‘Pulmonary embolism is characterized by sudden onset of chest pain, hemoptysis, and other discomforts’ we construct three matrices, as illustrated in Figure 6. Figure 6A represents the link between the head and tail of the entities through which the three entities of pulmonary embolism, chest pain, and hemoptysis can be identified. Figure 6B displays the link between the subject’s head and the objects’ head, revealing the relations between entities with specific head characteristics. The relation between the entity with ‘肺’ as the head of the entity and the entity with ‘胸’ as the head of the entity, and the entity with ‘肺’ as the head of the entity and the entity with ‘咳’ as the head of the entity can be learned from Figure 6B. Figure 6C demonstrates the link between the subject’s tail and the objects’ tail, revealing the relations between entities with particular tail features. We can learn from Figure 6C that the relation between the entity with ‘塞’ as the tail of the entity and the entity with ‘痛’ as the tail of the entity, and the entity with ‘塞’ as the tail of the entity and the entity with ‘血’ as the tail of the entity. By combining Figures 6B,C, we can learn the relations between pulmonary embolism and chest pain and pulmonary embolism and hemoptysis, with pulmonary embolism as the subject and chest pain and hemoptysis as the objects. This joint extraction method for entities and relations achieves simultaneous extraction while avoiding error propagation.

#### 2.2.4 Global pointer layer

The Global Pointer layer constructs global features directly using word vectors and fuses the global matrices to enhance the performance of model. After the word vector sequence is obtained, two feedforward neural networks are employed based on the span’s beginning and end indices. Similar to the BCsSa layer, this span is treated as entity head and entity tail, subject head and object head, and subject tail and object tail. Span boundary information is incorporated into rotational position encoding (RoPE) (30) to construct the global features.

#### 2.2.5 Multi-label cross-entropy loss

The electronic medical record dataset exhibits imbalances between positive and negative samples. The higher the degree of imbalance is, the more challenging it becomes to classify the data. In our joint extraction model, a large amount of redundant information is generated, exacerbating the degree of positive and negative sample imbalance and making data classification more difficult. Therefore, we employ the multi-label cross-entropy loss to mitigate the class imbalance problem and alleviate the effect of information redundancy on the model. The multi-label cross-entropy loss is expressed as equation (10).

(10) $$\mathrm{Loss}=\log\left(1+\underset{i\in P}{\sum }e^{-S_{i}}\right)+\log\left(1+\underset{i\in N}{\sum }e^{S_{i}}\right)$$

Where *P* is the positive sample set, *N* is the negative sample set, and *S_i_* represents the scores of the *i* class.

## 3 Results

### 3.1 Experimental setting

All training process was completed on a Windows 10 64GB RAM computer with an Intel(R) Core(TM) i7-10700F CPU @ 2.90GHz and a single RTX 3060 GPU 12GB RAM running on Python 3.7 and PyTorch 1.8.0.

In the experimental setup, we stipulated specific parameters to govern the training process. Specifically, we established a maximum sentence length of 256 tokens, a batch size of 16, a learning rate of 0.0001, and conducted training for 100 epochs. These parameters were carefully chosen to optimize model performance and facilitate comprehensive learning within the defined computational constraints.

### 3.2 Evaluation

The three most prevalent standard evaluation metrics consist Precision (P), Recall (R), and F1 score (F1), mathematically defined as Equations (11–13).

(11) $$P=\frac{TP}{TP+FP}$$

(12) $$R=\frac{TP}{TP+FN}$$

(13) $$F1=\frac{2PR}{P+R}$$

Where, TP (True Positive) represents instances where the positive class is accurately predicted as positive; FP (False Positive) refers to cases where the negative class is incorrectly predicted as positive; and FN (False Negative) signifies instances where the positive class is incorrectly predicted as negative. The term (TP + FP) reflects the proportion of correct predictions within the positive class results, and (TP + FN) indicates the proportion of actual positive class samples that are correctly classified as positive.

### 3.3 Experimental results and analysis

#### 3.3.1 Main results

To verify the performance of BCSLinker, we have the following four advanced baseline models involved in the comparison: SPN (21), CasRel (17), BiRTE (19), PRGC (18), OneRel (22), MultiHead (24), and GRTE (23).

**Figure 7:**
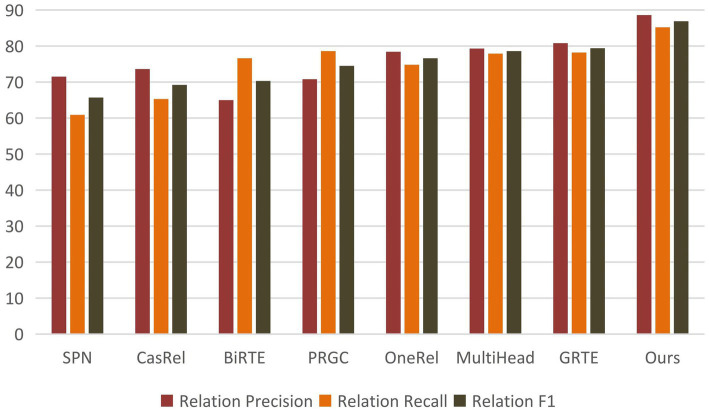
Histogram of the main results.

Table 4 demonstrates that BCSLinker achieved an F1 score of 86.9%, outperforming the other models. Figure 7 highlights that BCSLinker’s Precision, Recall, and F1 score on the electronic medical record dataset are significantly enhanced compared to the baseline models.

**Table 4:** Precision, recall and F1 score of our proposed BCSLinker and baselines.

**Model**	**Relation**		
	**Precision**	**Recall**	**F1**
SPN	71.5	60.9	65.7
CasRel	73.6	65.3	69.2
BiRTE	65.0	76.6	70.3
PRGC	70.8	78.6	74.5
OneRel	78.4	74.8	76.6
MultiHead	79.3	77.9	78.6
GRTE	80.8	78.2	79.4
Ours	**88.6**	**85.2**	**86.9**

The bold values represent the best results of the experiment.

The outstanding performance of BCSLinker can be attributed to two main advantages: First, we extract entities and relations simultaneously using a multi-module one-step extraction method, effectively alleviating error propagation. Second, we adopt the multi-label cross-entropy loss to mitigate the impact of negative samples, which widely exist in multi-module one-step extraction methods.

Baseline models, such as CasRel, BiRTE, and PRGC, extract entities and relations separately through multiple steps, essentially following pipelined extraction patterns and facing error propagation. As shown in Table 4, compared to the typical multi-module multi-step method PRGC, BCSLinker improves Precision, Recall, and F1 score on the electronic medical record dataset by 17.8, 6.6, and 12.4%, respectively, which suggests that simultaneously extracting entities and relations can mitigate error propagation.

SPN, OneRel, MultiHead, GRTE, and BCSLinker simultaneously extract entities and relations in one step. Although this method avoids error propagation, it generates substantial redundant information, leading to excess negative samples. The electronic medical record dataset inherently contains more negative samples, exacerbating the imbalance between positive and negative samples. Moreover, SPN exhibits a bias exposure problem. Table 4 reveals that BCSLinker, compared to the GRTE model, improves Precision, Recall, and F1 score by 8.2, 7, and 7.5%, respectively, on the electronic medical record dataset, which indicates that utilizing the multi-label cross-entropy loss suppresses the impact of information redundancy on the model, enhancing its performance.

#### 3.3.2 Ablation study

To evaluate the efficacy of each component, we remove particular component(s) at a time to assess the impact on the model. Table 5 reveals that: (1) Precision remains virtually unchanged when the common-sequence self-attention module is removed, while Recall and F1 score decrease by 1.1 and 0.5%, respectively, suggesting that common-sequence self-attention plays an essential role in extracting common features from the electronic medical record dataset. (2) When the BiGRU module is removed, the F1 score decreases by 1.2%, indicating that BiGRU’s extraction of contextual features significantly affects the performance of subsequent modules. (3) When both the BiGRU and the BCsSa are removed, the F1 score decreases substantially. Combined with the previous observations, this indicates that the BCsSa module effectively enhances feature interactions between medical entities in the electronic medical record text, thus improving the performance of BCSLinker. (4) When the Global Pointer module is removed, the F1 score decreases by 1.1%, suggesting that global features constructed by the Global Pointer module, combined with positional encoding, can compensate for shortcomings in the BCsSa module to some degree.

**Table 5:** An ablation study of BCSLinker on the dataset.

**Model structure**	**Relation**		
	**Precision**	**Recall**	**F1**
Ours	**88.6**	**85.2**	**86.9**
Remove common-sequence self-attention	88.5	84.3	86.4
Remove Global Pointer	87.7	83.9	85.8
Remove BiGRU	86.4	85.0	85.7
Remove BiGRU and BCsSa	86.6	82.3	84.4

The bold values represent the best results of the experiment.

To evaluate the efficacy of the multi-label cross-entropy loss in dealing with imbalances between positive and negative samples, we performed an ablation study using the electronic medical record dataset. In this study, we substituted the multi-label cross-entropy loss with binary cross-entropy loss (BCE) and compared the respective performance outcomes, as shown in Table 6. The results clearly indicate that the model’s performance, specifically with respect to Recall and F1 score, was compromised when the BCE was used. This establishes the superior effectiveness of the multi-label cross-entropy loss.

**Table 6:** The comparative evaluation of sample imbalance loss on the dataset.

**Model structure**	**Relation**		
	**Precision**	**Recall**	**F1**
Multi-label cross-entropy loss	**88.6**	**85.2**	**86.9**
BCE loss	87.9	78.3	82.8

The bold values represent the best results of the experiment.

#### 3.3.3 Storage and application of the knowledge graph

**Figure 8:**
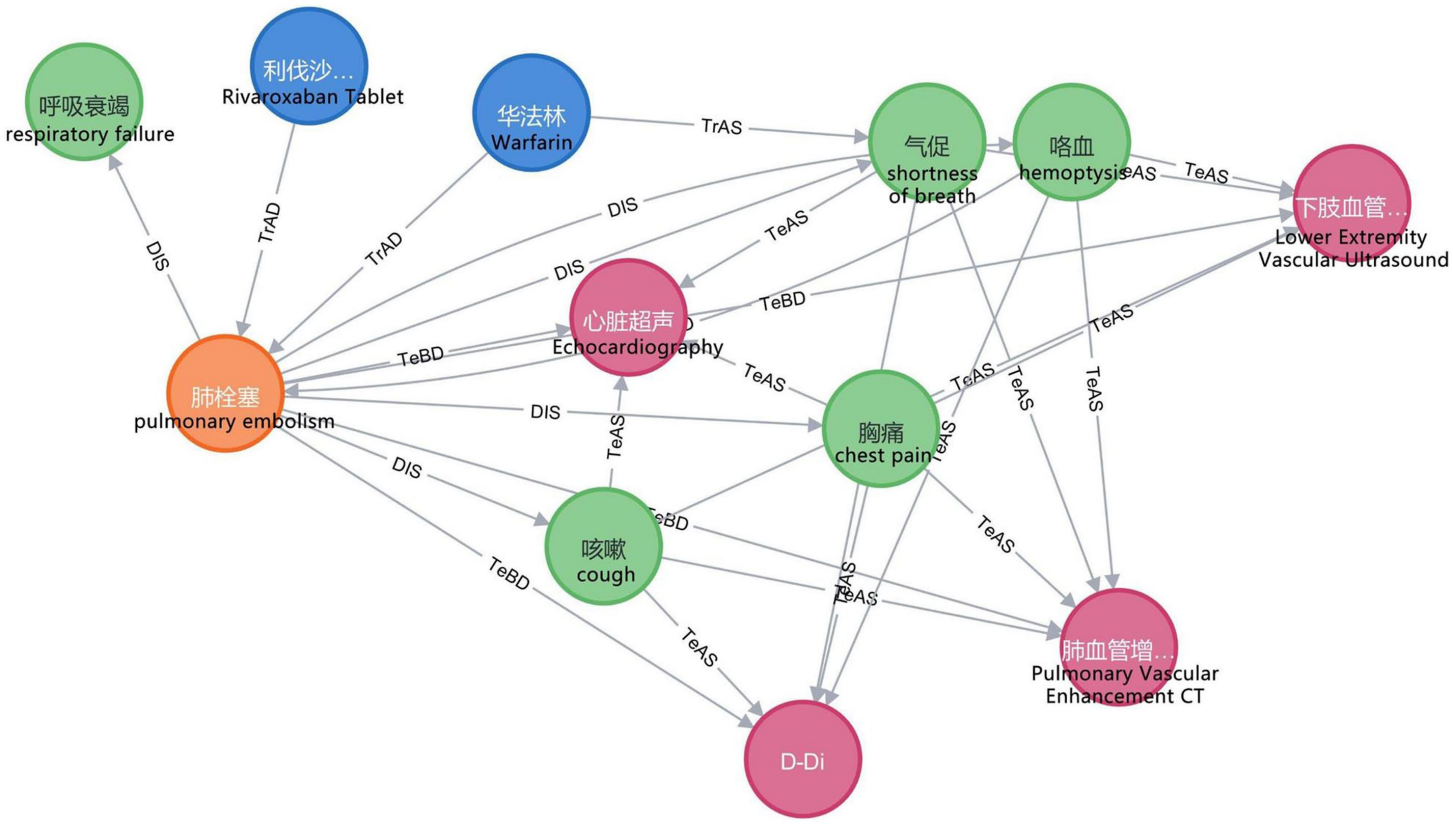
Part of the knowledge graph. To make them more understandable, we have provided the corresponding English explanations below each Chinese entity.

In this study, we employ the Neo4j graph database to store entities and relations and to map the VTEKG. The graph database differs from traditional relational databases because it stores ontologically structured knowledge and visualizes relations between entities. After extracting all relational triples in the electronic medical records using BCSLinker, we import the relational triples in batches from CSV files into the Neo4j graphical database, query the imported data using the Cypher language, and visualize the results. The Neo4j graph database enables searching and inference within the knowledge graph. Figure 8 demonstrates a small portion of our knowledge graph containing information about various types of medical entities and illustrating their semantic relations.

**Figure 9:**
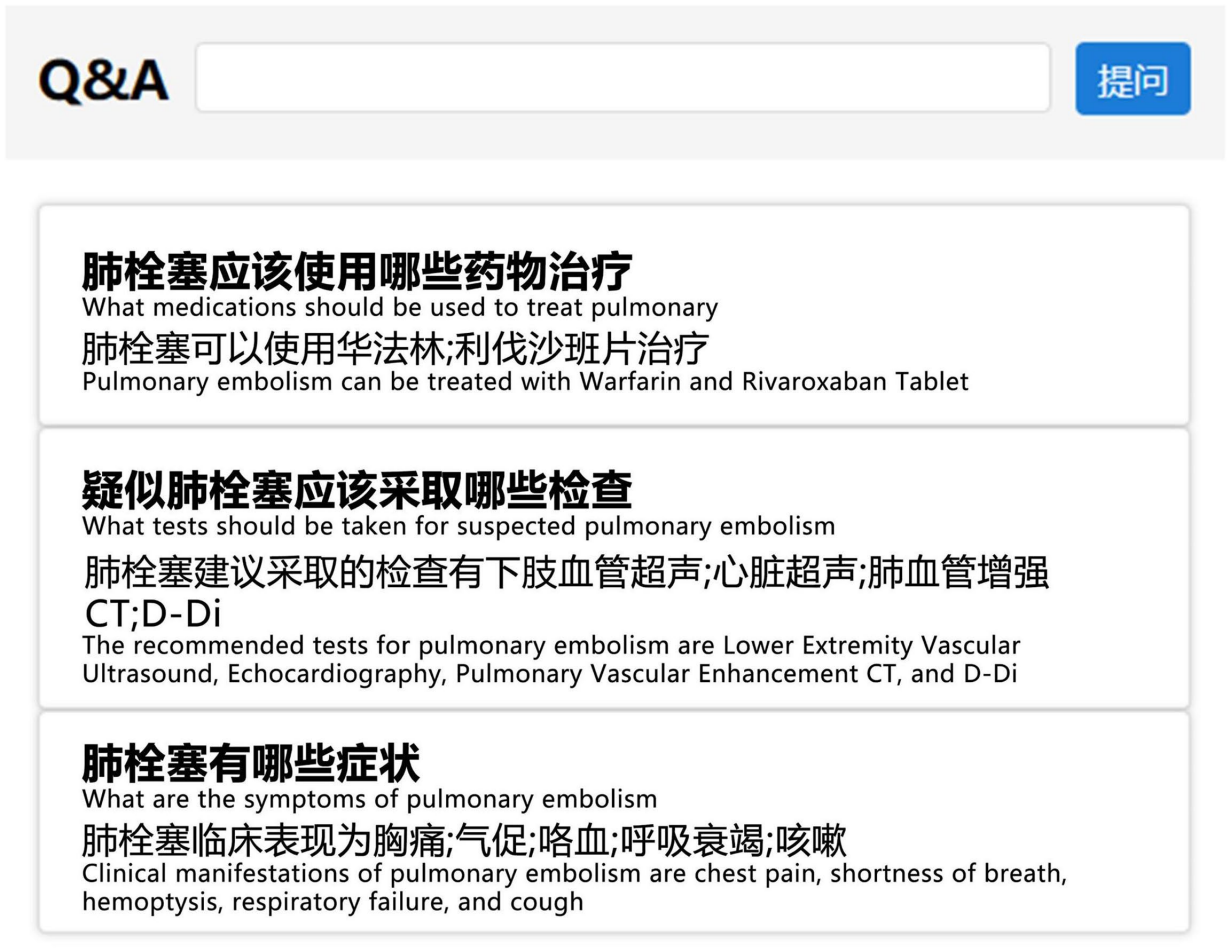
Example sentences from the question-answering system section. To make them more understandable, we have provided the corresponding English explanations below each Chinese sentence.

Upon completing the construction of VTEKG, we developed a question-answering system for VTE based on VTEKG, which supports queries for 14 types of questions. This system can serve as reference for VTE-related disease diagnosis, treatment, and patient self-care. The system first analyzes user questions, extracts relevant entities, and classifies the questions based on feature words. Then, the question is transformed into Cypher to find answers within the Neo4j graph database. Finally, the answers are combined with answer templates for related questions and returned to the user, facilitating human-computer interaction. Example sentences for three types of questions are shown in Figure 9.

In practical application, when a user inputs “What are the symptoms of pulmonary embolism?” into the VTE question-answering system, the system automatically identifies and extracts the entity “pulmonary embolism.” Furthermore, the system classifies the question under the DIS type based on the feature word. The question is subsequently translated into Cypher to search for nodes associated with a DIS relation to the “pulmonary embolism” node. As illustrated in Figure 8, nodes linked to “pulmonary embolism” via a DIS relation encompass “chest pain,” “shortness of breath,” “hemoptysis,” “respiratory failure,” and “cough.” Ultimately, leveraging predefined question-answering templates, the system responds: “Clinical manifestations of pulmonary embolism are chest pain, shortness of breath, hemoptysis, respiratory failure, and cough.”

## 4 Conclusion

In this study, we propose a joint entity and relation extraction model for constructing a VTE knowledge graph. This model utilizes the Biaffine Common-Sequence Self-Attention module to create global matrices, thus avoiding error propagation, and employs the multi-label cross-entropy loss to minimize the impact of redundant information. The experimental results show that Biaffine Common-Sequence Self-Attention Linker achieves a superior F1 score on the experimental dataset and more accurately and comprehensively detects patterns related to VTE and its associated diseases. Moreover, we use the VTEKG as a structured data source to develop an intelligent question-answering system, providing reference for diagnosis, treatment, and patient self-care for VTE and its related diseases.

In future research, we intend to augment the dataset size, thereby enhancing the efficacy of our model. Additionally, we will endeavor to incorporate a greater wealth of *a priori* information gleaned from medical guidelines. Furthermore, meticulous manual scrutiny and rectification of the knowledge graph will be undertaken to mitigate potential inaccuracies. Moreover, leveraging the VTE knowledge graph in tandem with the expansive language model, we aim to refine and extend the VTE question-answering system. This integration aims to ameliorate the interpretability shortfall inherent in large language models, thereby enhancing their utility, convenience, and precision in clinical contexts.

## Data availability statement

The data analyzed in this study is subject to the following licenses/restrictions: the datasets generated and analyzed during the current study are available from the corresponding author on reasonable request. Requests to access these datasets should be directed to JH, jfenghe@kmust.edu.cn.

## Ethics statement

Written informed consent was obtained from the individual(s) for the publication of any potentially identifiable images or data included in this article.

## Author contributions

FC: Writing – original draft, Conceptualization, Methodology, Formal Analysis. JH: Writing – review & editing, Conceptualization, Methodology, Project administration. YL: Writing – review & editing, Formal Analysis. HZ: Writing – review & editing, Formal Analysis.
